# ProteinTools: a toolkit to analyze protein structures

**DOI:** 10.1093/nar/gkab375

**Published:** 2021-05-21

**Authors:** Noelia Ferruz, Steffen Schmidt, Birte Höcker

**Affiliations:** Department of Biochemistry, University of Bayreuth, 95447 Bayreuth, Germany; Computational Biochemistry, University of Bayreuth, 95447 Bayreuth, Germany; Department of Biochemistry, University of Bayreuth, 95447 Bayreuth, Germany

## Abstract

The experimental characterization and computational prediction of protein structures has become increasingly rapid and precise. However, the analysis of protein structures often requires researchers to use several software packages or web servers, which complicates matters. To provide long-established structural analyses in a modern, easy-to-use interface, we implemented ProteinTools, a web server toolkit for protein structure analysis. ProteinTools gathers four applications so far, namely the identification of hydrophobic clusters, hydrogen bond networks, salt bridges, and contact maps. In all cases, the input data is a PDB identifier or an uploaded structure, whereas the output is an interactive dynamic web interface. Thanks to the modular nature of ProteinTools, the addition of new applications will become an easy task. Given the current need to have these tools in a single, fast, and interpretable interface, we believe that ProteinTools will become an essential toolkit for the wider protein research community. The web server is available at https://proteintools.uni-bayreuth.de.

## Graphical Abstract

**Graphical Abstract:**
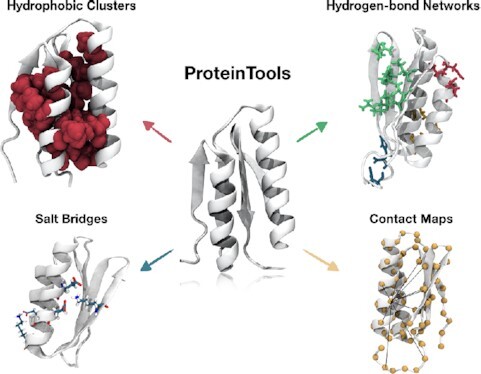
ProteinTools offers the analysis of hydrophobic clusters, hydrogen bonds, salt bridges, and contact maps in protein structures in an easy-to-use modern interface.

## INTRODUCTION

The number of deposited structures in the protein databank is growing at an exponential rate, with 90% of today's available structures deposited in the last 20 years. Not only provide experimental methods a wealth of structural data faster than ever before, but also computational efforts to predict structures have significantly advanced in the last decade. Particularly promising have been recent deep-learning-based methods on structural prediction, such as DMPfold (1) or AlphaFold (2). Efforts, both in experimental and computational fields, have enabled the characterization of protein structures at unprecedented speed and detail. Therefore, it is imperative that we implement computational tools to analyze these structures at comparable rates and to make such tools available to the broad community.

The 3D structure of a protein is important for its biological function, and therefore, its characterization or accurate prediction is of vital importance. Proteins fold into their native structures in an interplay driven by various non-covalent interactions such as hydrogen bonds, Van der Waal forces, hydrophobic, and ionic interactions. Thus, to understand a protein's features and functions at the molecular level, it is essential to characterize these interactions. While most computational efforts in structural biology have focused on implementing tools that predict protein structures, a few remarkable tools have also been released for structural analysis.

Many of these tools originated from the necessity to understand interactions in the context of protein dynamics (3) and thus focus on the analysis of molecular dynamic (MD) trajectories or are extensions of MD toolkits. Packages worth mentioning are Gromacs (4) and MDtraj (5), which enable the analysis of the time-evolution of molecular interactions in the command-line and Python languages. Other standalone packages focus on analyzing these interactions. In particular, the analysis of hydrogen bonds has attracted much attention since they play a major role in protein folding, structure, and function (6). Many tools that identify and analyze hydrogen bonds are available. To name a few, HBPredicT infers hydrogen bonds among water, ligands, and proteins (7). The molecular visualization programs Chimera (8), PyMOL (9) and VMD (10) all offer several tools to infer hydrogen bonds in proteins and ligands. An algorithm to plot hydrogen bonds in a global context, HBplot (11), was also recently developed.

Regarding the function of a protein, another property that is interesting to characterize is the detection and analysis of cavities and channels. Tools such as PASS (12) and PocketPicker (13), that both detect binding pockets, and CAVER (14), a web server for the visualization of catalytic pockets in proteins, have been developed. Many other tools focus on evaluating salt bridges, such as the web server ESBRI (15), or SBION (16), a program for the computation of salt bridges from multiple structure files.

Despite these significant advances, most of these tools offer an analysis of an individual structural property and are often available in software packages written in different programming languages. Therefore, users have to download and install several tools, and to consult various documentations. It is thus vital to improve such tools to make them usable in an intuitive manner. Particularly valuable are toolkits that gather many tools of interest in a single website, reducing users’ analysis times and learning curves. To our knowledge, not many web servers in the protein field have been published that collect several tools in a single site, although we expect this trend to change. We would like to highlight the Bioinformatics Toolkit (17) for the analysis of protein sequences: It includes among others remote homology detection, structure prediction, sequence alignments, and sequence clustering. The PlayMolecule toolkit (18), on the other hand, offers ligand-binding analysis, including tools such as ligand parameterization or prediction of binding affinities. Also, toolkits have been assembled to validate model quality, particularly of X-ray and NMR structures (19,20). Other past initiatives to implement toolboxes were the bPE toolkit (21), a toolkit for protein engineering and design, and StrucTools, that contained several tools such as the computation of Ramachandran plots or surface and volume calculations. These last two examples are unfortunately no longer maintained.

Motivated by the increased need for tools that analyze the growing wealth of structural data in a fast and self-contained manner, we developed ProteinTools (https://proteintools.uni-bayreuth.de), a toolkit for analyzing protein structures. At this stage we added four applications: The identification of hydrophobic clusters, hydrogen bond networks, salt bridges, and contact maps. Hydrophobic clusters prevent water molecules' intrusion into the protein core and serve as bodies of stability in high-energy partially folded states. Previous software and servers to compute hydrophobic clusters, such as the Contacts of Structural Units (CSU) algorithm (22) and the BASIC web server (23), are unfortunately no longer available. With the recent advent of powerful non-Adobe Flash/Java web molecular visualization tools such as the web app Mol* (https://molstar.org/), we can bring back the computation of hydrophobic clusters to the community. Hydrogen bond networks enable the communication between residues far apart in the protein structure (6,24). They help stabilize the protein and play a role in allostery. Despite their relatively easy identification, a web tool that analyzes and displays hydrogen bond networks is still missing. Other often requested analysis tools by protein researchers are the computation of salt bridges and contact maps. We have thus also included solutions to these problems in ProteinTools. To showcase the application of these four tools we use the domain Di-III_14 as an example, a designed IF-3 like fold with 74 amino acids that presents unusual folding properties (25,26).

## MATERIALS AND METHODS

### Hydrophobic clusters

It has been proposed that sidechains of isoleucine (ILE), leucine (LEU) and valine (VAL) residues often form hydrophobic or so-called (ILV)-clusters that prevent the intrusion of water molecules and serve as cores of stability in high-energy partially folded states (23). Various tools for the analysis of hydrophobic clusters solely from protein sequences have been developed (27) and recently made available as a Python package (28). Another possibility is to identify hydrophobic clusters directly in a protein structure. Their computation is based on the Contacts of Structural Units (CSU) algorithm, which is also widely used to calculate contact maps (22,29). Although the CSU algorithm was initially released as a package and web server, both are unfortunately no longer available. More recently, the CSU algorithm was applied to the particular case of computing contacts between hydrophobic atoms to define ILV clusters and it was released in the BASIC web server, which is also no longer accessible (23). The original algorithm operates as follows: Two atoms A and B are considered to be in contact if a solvent molecule placed at the surface of A’s sphere overlaps with the Van der Waals sphere of atom B plus the sphere formed by another solvent molecule (30). The atoms are considered spheres of fixed radius (31). If a water molecule penetrates several atoms' spheres at any position, the contact is considered to belong to the one whose center is closest to the center of atom A.

In practical terms, ProteinTools takes each ILE, VAL, and LEU heavy atoms into account and then retrieves the coordinates of their neighboring atoms. In case of alternate conformations only the first state is considered. These are atoms that are closer than the sum of the two Van der Waals radii, each enlarged by the water molecule radius (1.4 Å). Hence, for two carbon atoms to be considered candidates for atomic contacts, they must be within 6.56 Å. ProteinTools discretizes each atom sphere into 610 uniform sections using the Fibonacci grid (32,33). The area corresponds to a 0.0016th of the total area of the sphere. Then, the algorithm evaluates if any of the 610 sections overlap with its neighbors. If so, the section's contact is declared to belong to the atom whose center is closest to the center of the original sphere.

The algorithm is followed for all atoms until a matrix of residue-against-residue areas is computed. By default, ProteinTools defines that two residues are in contact when they have a total overlapping area of at least 10 Å^2^. The adjacent matrix is converted to a graph, where every component corresponds to a (hydrophobic) cluster. The cluster's total area is computed by the sum of the individual residue areas that comprise it. ProteinTools shows each of the computed hydrophobic clusters in a different color in an interactive panel. Properties of each cluster are summarized in a table. The results are available for download in the form of a PyMOL session (9) and a table. ProteinTools' implementation of hydrophobic clusters relies on the SciPy and NumPy Python packages.

### Hydrogen bond networks

Hydrogen bond networks are webs of hydrogen bonds that connect the sidechains of multiple residues across the protein. To compute the different hydrogen bond networks, ProteinTools first protonates the user-given coordinates with PROPKA (34) and PDB2PQR (35). For reproducibility, we (re)protonate all PDBs and only consider the first conformation of alternate sidechains. Following the PDB2PQR algorithm, protons are added after estimating pKa values for each residue at a pH of 7.0 (34). Also, sidechains are flipped and rotated to optimize local hydrogen bond networks (35). After protonation, ProteinTools computes all hydrogen networks in the protein sidechains using the Baker-Hubbard algorithm (36). We choose the cutoffs of ϑ > 120° and *d* < 2.5 Å, where ϑ is the angle defined by the three atoms and *d* is the distance between the donor hydrogen and the acceptor atom. ProteinTools backend relies on the MDtraj package for some of these computations (5). The atoms considered in this method are ‘NH’ and ‘OH’ as donors, and oxygen and nitrogen as acceptors. Once all hydrogen bonds have been computed, we consider any two residues as being connected if a consecutive path of hydrogen bonds between them can be found. ProteinTools assigns a different color to each network in the interactive Mol* panel. Each hydrogen bond is separately described in a table. Tables and protein structures can be downloaded as CSV files and PyMOL sessions (9), respectively.

### Salt bridge and charge distribution calculations

We determine salt bridge networks by selecting all acidic oxygen and all basic nitrogen atoms and computing an all-against-all matrix of their distances. Those pairs with distances below 4 Å are considered a salt-bridge. Alternate locations of sidechains are not considered, keeping in all cases only the first state. ProteinTools depicts each salt bridge cluster separately in an interactive window. This application also provides the computation of the κ (kappa) and Fraction of Charged Residues (FCR) parameters, primarily studied by the Pappu Lab (37). κ is a measure of the extent of charge segregation in a sequence. FCR is the fraction of charged residues in a sequence. These values can be used to predict the compactness of proteins. ProteinTools computes these values using the CIDER package (38). The protein structures with salt bridges visualized can also be downloaded as a PyMOL session (9).

### Contact maps

Protein contact maps represent distances between all amino acid residue pairs in the form of a matrix. ProteinTools calculates contact maps by computing an all-against-all distance matrix of residues and takes the minimum distance between any two atoms in the two evaluated residues. The raw data is plotted in an interactive panel, which can be exported as a CSV table.

### Implementation of protein tools

ProteinTools is developed using the Django Python framework (version 3.1.2). The backend is entirely implemented in Python. The website interface is designed with JavaScript using the Bootstrap framework (version 4.2). The proteins are visualized with the PDBe Molstar web package (39). Specific Python packages used on each application are cited in the above sections. All applications require a PDB code or a user-defined PDB structure as input and provide an interactive window as output. Data can be downloaded as CSV tables and for external visualization PyMOL sessions are provided when suitable. The web grants free access to all users and requires no login. Documentation is provided for each application separately in https://proteintools.uni-bayreuth.de.

## RESULTS

We demonstrate ProteinTools' four applications by using the protein Di-III_14 (PDB code 2LN3) as an example. In 2012, in an exceptional work by Koga *et al.* (26), rules were defined for the design of idealized protein structures and several protein folds found in nature were designed using these principles. Proteins designed in this work comprised the Ferredoxin-like fold, the Rossman 2 × 2 and 3 × 1 folds, the P-loop 2 × 2 fold, and the IF3-like fold. One of the IF3-like fold designs, Di-III_14, was further analyzed by Robert Matthews and his lab (25). Di-III_14 is a 74-amino acid long protein with four β-strands and two alpha-helices packed on one side of the β-sheet. The order of the β-strands is 1243 with 4 being antiparallel to the others. The researchers observed that although Di-III_14 unfolds in a two-state manner in the millisecond timescale, it remains folded several seconds in high concentrations of urea, which is an unusual feature among natural proteins. Experiments revealed numerous high-energy states that interconvert in slow timescales, which structurally corresponded to the formation of large electrostatic networks and hydrophobic clusters. Here, we use ProteinTools to show the computation of these properties.

### Di-III_14 contains a large hydrophobic cluster

**Figure 1.**
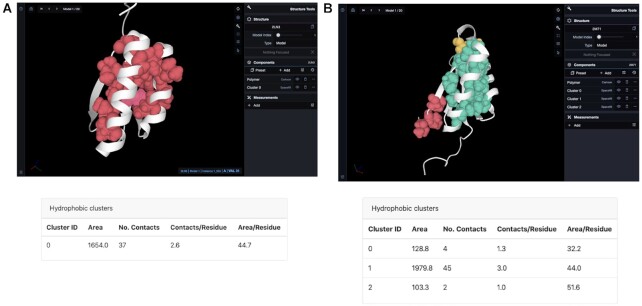
Hydrophobic cluster analysis of protein Di-III_14 (PDB 2LN3) (a) and another IF3-like natural protein (PDB 2M71) (B). (**A**) Di-III contains one larger hydrophobic cluster. A table with the summary of cluster properties is represented along with the structure. (https://proteintools.uni-bayreuth.de/clusters/structure/2ln3). (**B**) The IF3-like protein contains 3 hydrophobic clusters, with the largest cluster with an area of 1979.8 Å^2^**(**https://proteintools.uni-bayreuth.de/clusters/structure/2m71). Residues of a cluster get highlighted (pink) when mousing over them.

Basak *et al.* performed hydrogen exchange (HDX) NMR analyses of Di-III_14 that showed an exchange process between conformational states in slow timescales, and that stands in striking disparity with the fast process of unfolding revealed in guanidinium chloride denaturation (25). While both processes tend to provide comparable estimates in natural proteins, the stability optimization carried out during the protein design process can introduce multiple interactions that stabilize a tightly packed interior, leading to complex behaviors not observed in natural proteins. The authors mapped the strongly protected main chain amide hydrogens (NHs) onto the structure and found that they correspond to a large hydrophobic core surrounded by polar side chains. Here, we computed Di-III_14’s hydrophobic clusters to complement these results, an analysis that can be viewed at https://proteintools.uni-bayreuth.de/clusters/structure/2ln3 (Figure 1A). The analysis of hydrophobic clusters reveals a single cluster comprising 14 residues, with a total area of 1654.0 Å^2^. The cluster spans residues through all the secondary structure elements, with most amino acids belonging to the β-strands. The area per residue is 44.7 Å^2^, and there are 37 total contacts among the residues. We wondered whether these values correspond to an especially tightly packed IF3-like protein. We compared Di-III_14’s hydrophobic clusters with those of natural IF3-like proteins. To this end, we downloaded all domains from SCOPe (40), a database that classifies protein structures according to their topology and evolutionarily relationships. The SCOPe identifier for IF3-like proteins is d.68. After retrieving all proteins of the d.68 fold, we discarded those with sequence lengths over 150 amino acids, leading to 43 members outlined in Supplementary Table S1. Visualization of these structures with ProteinTools revealed an average cluster number of 2.2 per structure, with the cluster located between the helices and strands being the largest one in all cases. The average area for this cluster among the proteins is 1957.5 Å^2^, slightly larger than that in Di-III_14 (1654.0 Å^2^), but well within one standard deviation (±1078.9 Å^2^). The average residue number is 14.1, in line with the results for Di-III_14. A representative IF3-like protein with three clusters and an area of 1978 Å^2^ for the largest cluster is shown in Figure 1b for comparison. In light of these results, we cannot conclude that Di-III_14’s hydrophobic cluster differs significantly from those in IF3-like natural proteins.

### Hydrogen bond networks

**Figure 2.**
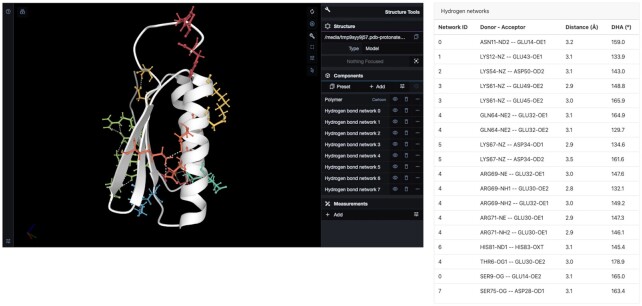
Hydrogen bond network analysis of protein Di-III_14 (PDB 2LN3). Next to the viewer window a table with the details for each hydrogen bond is given, including the network they belong to (https://proteintools.uni-bayreuth.de/bonds/structure/2ln3).

Basak *et al.* observed two electrostatic networks, one spanning α1 and α2’s surfaces and the other containing a quartet of salt bridges that link the two internal β-strands, β2 and β4. To recapitulate these findings, we computed Di-III_14’s hydrogen bond networks (https://proteintools.uni-bayreuth.de/bonds/structure/2ln3). ProteinTools computes hydrogen bond networks among sidechains by looking at nitrogen and oxygen donors and acceptors within 2.5 Å and an angle over 120° (see Materials and Methods). Di-III_14 contains eight hydrogen bond networks (Figure 2). The largest one, similar to the description by Basak *et al.*, spans β1, β2, and β4 and contains six residues (hydrogen bond network 4, Figure 2, light green). The residues are Thr6, Glu30, Glu32, Gln64, Arg69 and Arg71. Another two networks reinforce the internal strands' interactions: Network 5 (blue, Asp34 and Lys67) and network 7 (dark yellow, Asp28 and Ser 75).

In our analysis, we observe a total of four hydrogen bond networks in the helices, with three of them mostly spanning α2. Network 3 (orange) comprises residues Glu 45, Glu49 and Lys61 and brings together α2 and β3. Similarly, network 0 (red) links strands and helices by networking Ser9 in β1 with Asn11 and Glu14 in α1. The other two networks correspond to network 2 (yellow), entirely contained in α2 (residues Asp50 and Lys 54) and network 1 (dark green), linking α1 and α2 via Lys12 and Glu43.

### Salt bridges

**Figure 3.**
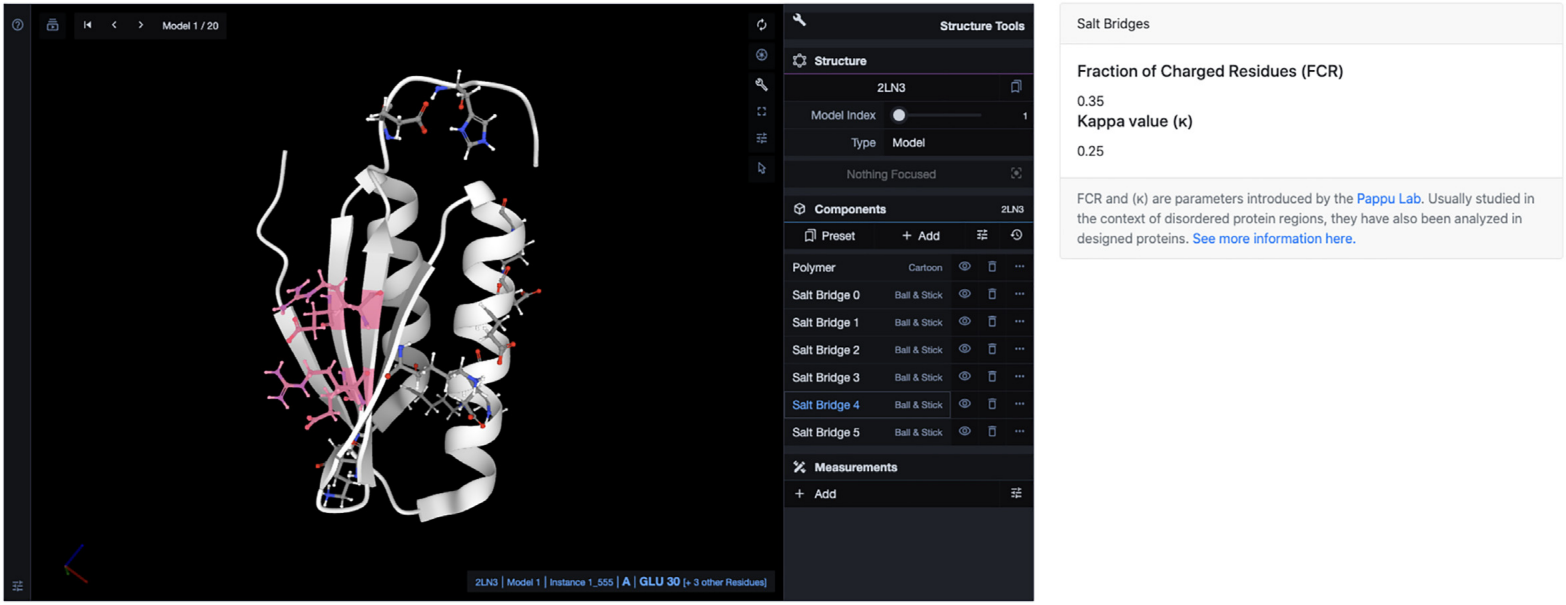
Salt bridge networks in protein Di-III_14 (PDB 2LN3). Residues get highlighted by mousing over them, in this case the depicted salt bridge 4. The κ and FCR parameters are shown on the right (https://proteintools.uni-bayreuth.de/salt/structure/2ln3).

ProteinTool's salt bridge application enables finding salt bridge networks in a protein and computing charge segregation parameters (37). We computed Di-III_14’s salt bridges at https://proteintools.uni-bayreuth.de/salt/structure/2ln3 (Figure 3). Di-III_14 has six salt bridge networks. The largest one, salt bridge 4 (highlighted), comprises many of the residues in hydrogen bond network 4: Glu30, Glu32, Arg69, and Arg71, and along with salt bridge 3 (Lys67 and Asp34), spans the internal β-sheet. Salt bridge 2 links the elements β4 and α2 (Lys61, Glu45, and Glu49), whereas salt bridge 0 links α1 and α2 (Lys12, Glu13, Glu40 and Glu43). Lastly, salt bridge 1, with residues Asp50, Lys53 and Lys54, spans one half of α2. Our networks agree with Basak *et al.*, with a few differences arising from our more stringent cutoff of a 4 Å distance between residue pairs.

Basak *et al.* suggested that the unusually large composition of charged sidechains differentiates the folding mechanism of DI-III_14 from natural proteins. The authors plotted the fraction of charged residues (FCR) versus κ for almost the entire proteome of the thermophile *Sulfolobus solfataricus* and observed that Di-III_14 appears at a different region than the rest of the proteins. ProteinTools is also capable of computing these parameters, giving an FCR of 0.35 and a κ of 0.25, in agreement with Basak *et al.*’s results. We wondered whether this differences between Di-III_14 and natural proteins also extends to the other designs in the work by Koga *et al.* (21). To this end, we took all SCOPe protein sequences from the corresponding folds in Koga *et al.*’s work and compared them with the designs. The designed folds and their SCOPe identifiers are: Fold-I: Ferredoxin-like fold (d.58), Fold-II: Rossmann 2 × 2 (c.2), Fold-III: IF3-like fold (d.68), Fold IV: P-loop 2 × 2 fold (c.37), Fold V: Rossmann 3 × 1 (c.23). The natural proteins belonging to these folds tend to present FCR values around 0.25 and κ values around 0.2 clustering in a similar region in space (Supplementary Figure S1a). The designed proteins, however, tend to have greater FCR values (FCR ≥ 0.35 in 4/5 cases) and lower κ values (κ = 0.12–0.14 in 4/5 cases) and therefore appear in the periphery (Supplementary Figure S1b). This effect could be due to an excessive stabilization via the introduction of interactions during the protein design process to ensure stable designs, but this hypothesis requires further investigation.

### Contact maps

**Figure 4.**
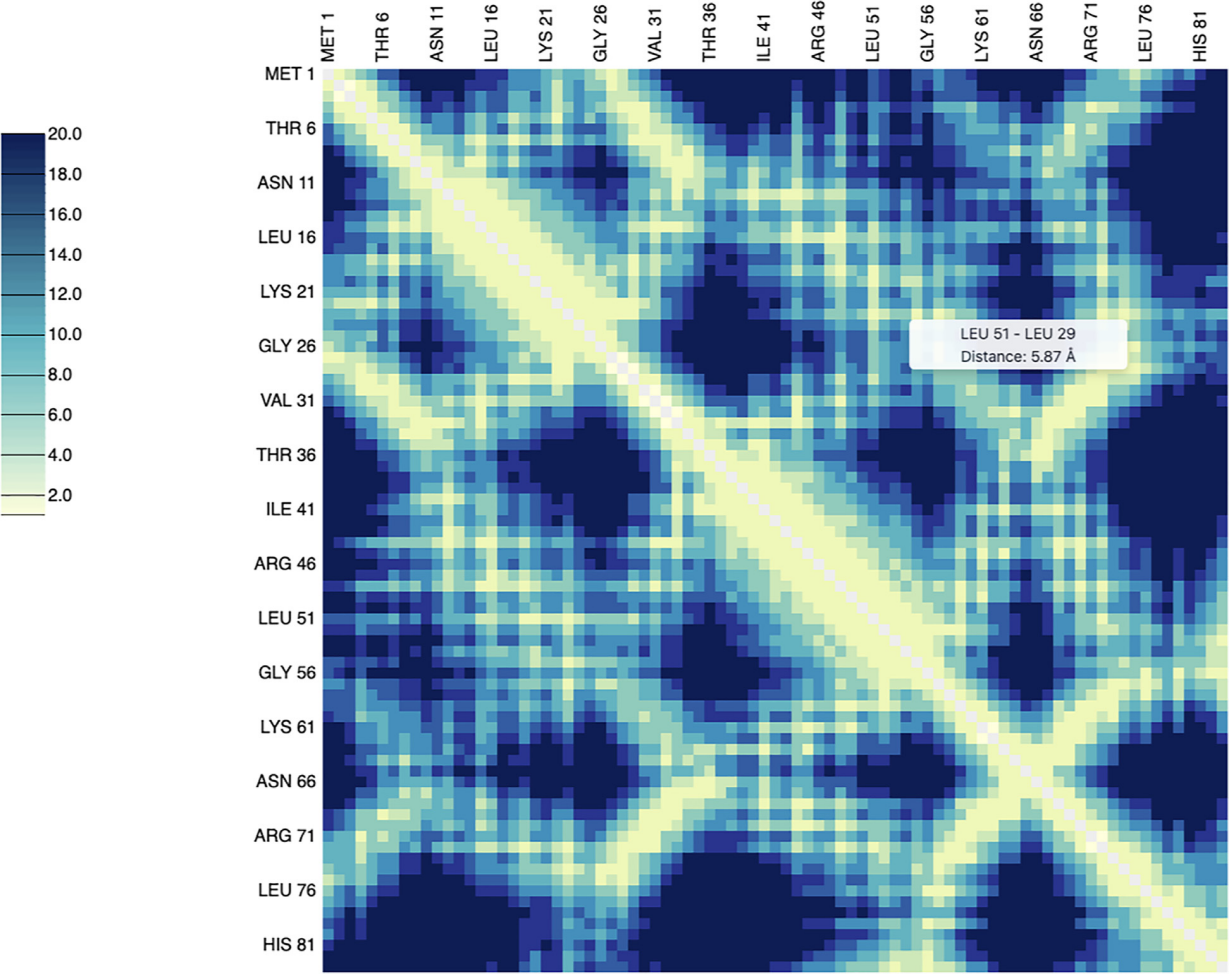
Contact map calculation for protein Di-III_14 (PDB 2LN3). A tooltip with involved residues and distances shows up when mousing over their corresponding position in the matrix (https://proteintools.uni-bayreuth.de/contacts/structure/2ln3).

Protein contact maps represent the distance between all possible amino acid pairs and provide a reduced representation of protein structures that is invariant to rotations and translations. They have been widely used in machine learning methods and can be applied to reconstruct 3D structures (41) or in protein similarity analysis (42). Therefore, a quick computation of contact maps is useful for a wide variety of purposes. As an example, we computed Di-III_14’s contact map (Figure 4).

## DISCUSSION

While new techniques and the automatization of processes are revolutionizing the generation of protein structural data, there is much need to also adapt the tools for their analysis. Web applications have become particularly useful in the last years: they (i) do not require installation, (ii) are accessible from any internet-connected computer, and (iii) liberate the user from learning specific programs. Among web servers, toolkits are particularly valuable as they gather several applications that would otherwise require various packages or web servers. These toolkits not only ease the use, but also help to guide the analysis and to view protein structures in a more complete manner and reveal common patterns (43). Motivated by these current needs, we implemented ProteinTools as a modular toolkit to analyze protein structures. So far, we implemented four much needed analysis tools: hydrophobic clusters, hydrogen bond networks, salt bridges, and contact maps. Its release is particularly timely and useful for the community, given that to our knowledge no other web server for the computation of hydrophobic clusters and hydrogen bond networks are currently available. The toolkit's modular nature will make the addition of other applications to ProteinTools easy. We envision integrating an application for the generation of mutants and estimating their ΔΔG°, as well as the computation of cavities in the near future. Given the current need for tools that analyze the growing number of protein structures and the opportunities for extending them, we strongly believe that ProteinTools will become an indispensable toolkit for the protein research community.

## DATA AVAILABILITY

The web server is available at https://proteintools.uni-bayreuth.de.

## Supplementary Material

gkab375_Supplemental_File
