# Supplementary material for: ProteinTools: a toolkit to analyze protein structures
Source: Nucleic Acids Res. 2021 May 21;49(W1):W559–66. doi: 10.1093/nar/gkab375 (PMC8262690; doi:10.1093/nar/gkab375)
Supplement: gkab375_Supplemental_File [file gkab375_supplemental_file.pdf]

Supporting Information for:

ProteinTools: A Toolkit to Analyze Protein Structures

Noelia Ferruz, Steffen Schmidt and Birte Höcker

<u>Content</u>

Table S1: Comparison of hydrophobic clusters for IF3-like proteins

Figure S1: Distribution of the normalized charge segregation parameter, k versus the fraction of charged residues (FCR) for *de novo* designs compared to natural proteins.

Table S1: Comparison of hydrophobic clusters for IF3-like proteins (SCOPe fold d.68). Hydrophobic clusters were compared for 43 protein structures. The number of clusters and area of the main cluster is outlined below. This information can be individually accessed at proteintools.uni-bayreuth.de/xxxx where xxxx is the corresponding PDB identifier.

PDB	SCOPe domain	No. of clusters	Area main cluster (Å^2)	No. of residues in main cluster
1DCJ	d1dcja_	3	1009.1	7
1H0X	d1h0xa_	2	3687.0	24
1H0Y	d1h0ya_	3	3132.7	21
1H4Q	d1h4qa3	3	100.4	2
1H4S	d1h4sa3	3	104.4	2
1H4T	d1h4ta3	2	101.3	2
1HC7	d1hc7a3	3	80.9	2
1JDQ	d1jdqa1	3	672.0	9
1JE3	d1je3a1	3	913.8	6
1JO0	d1jo0a_	2	2835.3	17
1LN4	d1ln4a1	2	3144.5	18
1MSZ	d1msza_	1	717.6	7
1NFH	d1nfha_	3	2323.3	20
1NFJ	d1nfja_	3	2488.9	21
1NH9	d1nh9a_	2	3249.8	21
1NJ1	d1nj1a2	2	1211.0	10
1NJ2	d1nj2a2	2	1230.0	10
1NJ5	d1nj5a2	2	1307.8	10
1NJ6	d1nj6a2	2	1255.3	10
1NJ8	d1nj8a2	2	2418.0	13
1PAV	d1pava_	2	682.4	6
1RQ8	d1rq8a_	2	2620.5	14
1TIG	d1tiga_	1	1706.8	15
1UDV	d1udva_	2	3140.3	22
1UG8	d1ug8a1	2	2151.6	13
1VM0	d1vm0a_	2	2540.1	17
1Y9X	d1y9xa_	2	2624.3	21
2A2Y	d2a2ya_	2	1783.7	17
2BKY	d2bkya_	3	3625.2	24
2D9I	d2d9ia1	3	1966.7	15
2H9U	d2h9ua_	1	3344.4	21
2IFE	d2ifea_	2	1978.5	14
2LRR	d2lrra_	1	745.1	8
2M71	d2m71a1	3	1979.8	15
2Q3V	d2q3va_	2	2422.0	17
2Z7C	d2z7ca_	1	2780.3	22
3FAU	d3faua1	1	2480.5	17
3LVJ	d3lvjc_	1	1097.1	9
3LVK	d3lvkb_	2	1006.2	8
3TOE	d3toea_	3	3005.5	21
3U6Y	d3u6ya_	2	3348.6	22
3WBM	d3wbma_	3	3539.4	23
4Z9E	d4z9ea_	5	1619.2	13
**Average**		2.2	1957.5	14.1

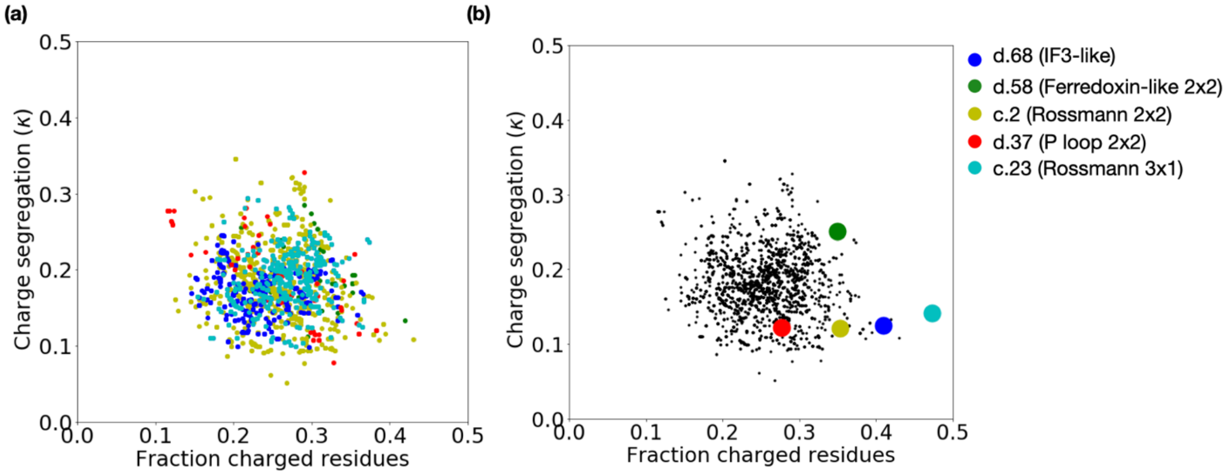

Figure S1: Distribution of the normalized charge segregation parameter, κ versus the fraction of charged residues (FCR) for *de novo* designs compared to natural proteins. (a) Superimposition of the values for all domains in SCOPe belonging to each of the corresponding folds (colors described in the legend). Only proteins with sequences below 150 amino acids in length were selected for the analysis to be in agreement with the designs' short lengths. **(b)** Comparison of all natural domains from the five folds (black) vs. the five designs from Koga et al.
